# Characterization of multidrug-resistant ST978-KL103 *Klebsiella quasipneumoniae* subsp. *quasipneumoniae* clinical strains co-harboring *tmexCD2-toprJ2* and *bla*_IMP-4_

**DOI:** 10.1128/spectrum.02622-25

**Published:** 2025-12-19

**Authors:** Jiale Wu, Si Xu, Zhiyou Xiao, Jiancong He, Derong Xu

**Affiliations:** 1 Department of Medical Instruments, The First Affiliated Hospital, Jiangxi Medical College, Nanchang University 47861, Nanchang, Jiangxi, China; 2 Jiangxi Institute of Translational Medicine, The First Affiliated Hospital, Jiangxi Medical College, Nanchang University 47861, Nanchang, Jiangxi, China; 3 Jiangxi Key Laboratory of Drug Target Discovery and Validation, Jiangxi Medical College, Nanchang University, Jiangxi, China; University of Pretoria, Pretoria, Gauteng, South Africa

**Keywords:** *Klebsiella quasipneumoniae *subsp. *quasipneumoniae*, ST978-KL103, virulence, resistance, *tmexCD2-toprJ2*

## Abstract

*Klebsiella quasipneumoniae* is an emerging opportunistic pathogen increasingly associated with multidrug-resistant infections. In this study, we characterized four clinical isolates of *K. quasipneumoniae* subsp. *quasipneumoniae* assigned to the ST978-KL103 clonotype, all linked to poor clinical outcomes. Antimicrobial susceptibility testing revealed an extensively drug-resistant phenotype, including resistance to β-lactams, carbapenems, aminoglycosides, fluoroquinolones, tigecycline, and trimethoprim/sulfamethoxazole, with polymyxin B as the only effective agent. Whole-genome sequencing and comparative analysis showed that all ST978-KL103 isolates harbored multiple plasmids, including a conserved IncU-type plasmid carrying *bla*_IMP-4_ and the *tmexCD2-toprJ2* tigecycline resistance cluster. Two isolates also carried IncC-type plasmids encoding *bla*_NDM-1_. Conjugation assays confirmed efficient horizontal transfer of both plasmid types to *E. coli* recipients. Although classical virulence loci such as yersiniabactin, aerobactin, and *rmpA*/*rmpA2* were absent, the isolates exhibited strong biofilm formation and serum resistance, indicating potential for persistence and immune evasion. Cytotoxicity assays and *Galleria mellonella* infection models demonstrated low acute virulence compared to the hypervirulent *K. pneumoniae* strain NUTH-K2044. Phylogenetic analysis placed the ST978 isolates within a distinct and globally distributed clonal lineage. The close genetic relatedness among isolates, particularly between KP57 and KP58, suggests recent nosocomial transmission. Resistome profiling of nine global ST978 genomes revealed over 40 resistance genes, with our isolates uniquely harboring the *tmexCD2-toprJ2* cluster. These findings highlight ST978 *K. quasipneumoniae* as a globally disseminated and increasingly drug-resistant lineage with the potential to emerge as a high-risk clone in clinical settings.

**IMPORTANCE:**

The emergence of extensively drug-resistant *Klebsiella quasipneumoniae* strains poses a significant threat to global health yet remains underrecognized due to diagnostic limitations. Our study identifies ST978-KL103 as a cryptic high-risk lineage co-harboring *bla*_IMP-4_, *bla*_NDM-1_, and tmexCD2-toprJ2 on transferable plasmids. These findings highlight the species’ underappreciated role as a reservoir and disseminator of last-line resistance. The strong biofilm formation and serum resistance further suggest persistence potential in clinical settings. Early recognition and surveillance of such emerging clones are critical to prevent silent dissemination and therapeutic failure in healthcare environments.

## INTRODUCTION

The rapid emergence and global spread of multidrug-resistant (MDR) *Klebsiella* species have become a critical public health concern, particularly in healthcare-associated infections characterized by high morbidity and mortality (1, 2). While *Klebsiella pneumoniae* remains the most frequently identified species within the genus, increasing attention has been directed toward closely related species such as *K. quasipneumoniae* (3, 4). These species are often misidentified in clinical diagnostics due to their high genomic similarity (5). Emerging evidence indicates that *K. quasipneumoniae* is capable of acquiring diverse carbapenemase plasmids from other bacterial species (4, 6).

*K. quasipneumoniae* subsp. *quasipneumoniae* has been detected in both clinical and environmental settings, including hospital sinks and wastewater systems, suggesting its ability to persist and disseminate in nosocomial environments (4, 7). Although infections caused by this subspecies are less frequently reported than those caused by *K. pneumoniae*, they involve similar clinical manifestations, including bloodstream and urinary tract infections (8, 9). Notably, *K. quasipneumoniae* has been found to harbor critical resistance genes, including *bla*_NDM_, *bla*_KPC_, and *bla*_OXA_, often located on mobile genetic elements (10–12). The convergence of resistance and persistence traits in this organism, combined with frequent misidentification, complicates infection control and therapeutic decision-making and underscores the need for more precise characterization (11).

Of particular concern is the emergence of plasmid-mediated resistance mechanisms that compromise the efficacy of last-resort antibiotics (13, 14). The *tmexCD-toprJ* efflux pump gene cluster, recently identified in *Klebsiella* species, confers transferable resistance to tigecycline, a key therapeutic agent against carbapenem-resistant Enterobacterales (15–18). The coexistence of *tmexCD-toprJ* variants with carbapenemase genes on conjugative plasmids poses the risk of pan-drug resistance and rapid horizontal gene transfer within and across species boundaries (19, 20). Despite these emerging threats, genomic and phenotypic studies focusing on *K. quasipneumoniae*, particularly strains that co-harbor multiple high-risk resistance determinants, remain scarce, leaving a significant gap in our understanding of its clinical and epidemiological significance.

In this study, we investigated four clinical isolates of *K. quasipneumoniae* subsp. *quasipneumoniae* belonging to the ST978-KL103 clonotype, all exhibiting extensively drug-resistant (XDR) phenotypes and associated with adverse clinical outcomes. Using whole-genome sequencing, conjugation experiments, virulence assays, and phylogenetic analysis, we aimed to elucidate the mobile genetic elements, resistance mechanisms, and virulence traits of these isolates. Our findings highlight the emergence of a genetically cohesive and globally distributed clone with the potential to pose a significant clinical and public health threat.

## MATERIALS AND METHODS

### Isolate identification and culture

Between January and May 2024, four *K. quasipneumoniae* subsp. *quasipneumoniae* isolates (KP55, KP57, KP58, and KP79) were obtained from clinical specimens collected from hospitalized patients in the Department of Clinical Laboratory, the First Affiliated Hospital of Nanchang University (Jiangxi, China). The clinical sources of the isolates included blood, sputum, central venous catheter tip, and cerebrospinal fluid. All isolates were initially cultured on MacConkey agar and blood agar plates and incubated aerobically at 37°C for 18–24 h. Preliminary species identification was conducted using matrix-assisted laser desorption ionization-time of flight mass spectrometry (MALDI-TOF MS; Bruker Biotyper, Bruker Daltonics, Germany). The isolates were subsequently stored in 30% glycerol stocks at –80°C for long-term preservation and further analysis.

### Antimicrobial susceptibility testing

Antimicrobial susceptibility testing was performed using the broth microdilution method with commercially available testing panels (Autobio, China), following the manufacturer’s instructions. The isolates KP55, KP57, KP58, and KP79 were subcultured on blood agar plates and incubated aerobically at 37°C overnight prior to testing. Susceptibility was assessed against a panel of antibiotics, including amikacin, ampicillin/sulbactam, azithromycin, ceftazidime, ceftriaxone, cefuroxime, ceftazidime–avibactam, gentamicin, imipenem, levofloxacin, meropenem, moxifloxacin, piperacillin–tazobactam, trimethoprim/sulfamethoxazole, tigecycline, and polymyxin B. MICs were interpreted according to the Clinical and Laboratory Standards Institute guidelines (2024), except for tigecycline and polymyxin B, which were interpreted based on breakpoints established by the U.S. Food and Drug Administration and the European Committee on Antimicrobial Susceptibility Testing, respectively. *K. quasipneumoniae* subsp. *similipneumoniae* ATCC 700603 and *E. coli* ATCC 25922 were used as the quality control strain. All susceptibility tests were performed in triplicate to ensure reproducibility.

### Whole-genome sequencing and analysis

Genomic DNA was extracted from cultured ST978 *K. quasipneumoniae* subsp. *quasipneumoniae* isolates using a bacterial genomic DNA extraction kit (Sangon Biotech Co., Ltd., Shanghai, China), following the manufacturer’s protocol. The concentration and purity of DNA were assessed using a NanoDrop spectrophotometer and Qubit fluorometer to ensure suitability for sequencing. Whole-genome sequencing was performed using the Illumina NovaSeq 6000 and the PacBio platform. Raw reads were quality filtered using fastp v0.20.1 to remove adapter sequences, low-quality bases, and short reads (21). Genome assembly was performed using Unicycler v0.4.8 (22). Annotation was carried out using Prokka v1.4.5 (23). Species confirmation and genotypic characterization were performed using Kleborate v3 (http://github.com/katholt/Kleborate). Plasmid replicon types were identified using the PlasmidFinder database, applying thresholds of ≥90% identity and coverage (24). To further investigate the plasmid content and mobility, Mob-Suite v2.1.0 was employed for plasmid classification (25). The detection of resistance and virulence genes was also carried out using Abricate (https://github.com/tseemann/abricate), with searches performed against the ResFinder database (https://bitbucket.org/genomicepidemiology/resfinder_db) and Virulence Factor Database (https://www.mgc.ac.cn/VFs/search_VFs.htm).

### Biofilm formation assay

*K. quasipneumoniae* subsp. *quasipneumoniae* cultures were grown overnight in Luria-Bertani (LB) broth at 37°C with shaking at 220 rpm. A total of 200 µL of each bacterial suspension was then transferred into individual wells of sterile 96-well polystyrene microtiter plates and incubated statically at 37°C for 24 h. After incubation, the planktonic cells were carefully removed, and the wells were gently washed three times with sterile phosphate-buffered saline (PBS) to remove non-adherent cells. The adherent biofilms were fixed with 99% methanol and subsequently stained with 1% (wt/vol) crystal violet for 10 minutes. Excess stain was removed by washing once with running tap water, and the plates were air-dried at 37°C. The biofilm biomass was quantified by measuring the optical density at 600 nm (OD_600_) using a microplate reader. The hypervirulent *K. pneumoniae* strain NTUH-K2044 and *K. quasipneumoniae* subsp. *similipneumoniae* strain ATCC 700603 were used as positive controls, while sterile LB broth served as the negative control. All assays were performed in triplicate and repeated three independent times to ensure reproducibility and consistency of the results.

### Serum killing assay

Overnight bacterial cultures were diluted and grown to mid-logarithmic phase, after which 25 µL of each bacterial suspension (approximately 1 × 10^6^ CFU) was combined with 75  µL of pooled human serum in sterile microcentrifuge tubes. The reaction mixtures were incubated at 37°C, and samples were retrieved at 0, 1, 2, and 3 h post-incubation to determine bacterial viability. Serial dilutions of each sample were plated onto Mueller–Hinton agar and incubated at 37°C for 24 h to enumerate CFUs. All experiments were carried out in triplicate. The survival rates of the clinical *K. quasipneumoniae* isolates were compared with those of the *K. quasipneumoniae* subsp. *similipneumoniae* strain ATCC 700603 and the hypervirulent strain NTUH-K2044 to assess relative serum resistance.

### Cytotoxicity assay

To evaluate the cytotoxic potential of *K. quasipneumoniae* subsp. *quasipneumoniae* isolates, a lactate dehydrogenase (LDH) release assay was performed using human alveolar epithelial A549 cells. A549 cells were maintained in Dulbecco’s modified Eagle’s medium (DMEM) supplemented with 10% heat-inactivated fetal bovine serum and 1% penicillin-streptomycin and incubated at 37°C in a humidified atmosphere containing 5% CO_2_. For infection experiments, cells were seeded into sterile 24-well tissue culture plates at a density of 1 × 10^5^ cells per well and allowed to adhere for 24 h until ~80% confluence. Prior to bacterial infection, the culture medium was replaced with antibiotic-free DMEM. Overnight-grown bacterial cultures were washed and resuspended in PBS and adjusted to a multiplicity of infection of 200:1 (approximately 2 × 10^7^ CFU per well). The bacterial suspension was added to each well, and the plates were incubated at 37°C for 6 h under standard conditions. After incubation, cell culture plates were centrifuged at 3,000 *g* for 5 minutes at 4°C to pellet cellular debris and bacteria. The supernatants were carefully collected and analyzed for LDH activity using the LDH Cytotoxicity Assay Kit (Solarbio, BC0685, China), following the manufacturer’s instructions. The absorbance was measured at 490 nm using a microplate reader, with background absorbance subtracted from all values. All assays were performed in triplicate wells and repeated in three independent experiments to ensure reproducibility.

### *Galleria mellonella* infection model

*G. mellonella* larvae weighing approximately 250–300 mg were obtained from Tianjin Huiyude Biotechnology Co., Ltd. (Tianjin, China). Larvae exhibiting signs of melanization, lethargy, or physical damage were excluded. Prior to injection, larvae were randomly divided into experimental groups. Bacterial suspensions of *K. quasipneumoniae* (approximately 1 × 10^6^ CFU per larva) were prepared, and 10  µL of each suspension was injected into the last left proleg using a Hamilton microsyringe. Control larvae received an equal volume of sterile 0.9% (wt/vol) saline solution under identical conditions. Following inoculation, larvae were transferred to sterile Petri dishes lined with clean filter paper and incubated at 37°C in the dark. The survival status of the larvae was assessed every 12 h for a total duration of 72 h. Death was defined by the appearance of complete melanization and the absence of a response to gentle tactile stimulation. Each experimental condition included 10 larvae, and all infections were conducted in three independent biological replicates to ensure reproducibility.

### Conjugation assay

Conjugation experiments were conducted to assess the ability of ST978-KL103 *K. quasipneumoniae* donor strains to transfer resistance plasmids to *E. coli* J53, a sodium azide-resistant recipient strain. Donor and recipient cultures were cultivated in LB broth at 37°C with shaking until reaching mid-logarithmic phase. Equal volumes of both cultures were mixed at a 1:1 ratio and centrifuged. The cell pellet obtained was carefully resuspended in MgSO_4_ solution and spotted onto LB agar plates. The plates were then incubated at 37°C overnight to facilitate conjugative transfer. After incubation, the mating mixtures were scraped from the plate surface, suspended in sterile saline, serially diluted, and plated onto selective LB agar containing appropriate antibiotic combinations to isolate transconjugants. For plasmids pKP55-1, pKP57-1, pKP58-1, and pKP79-1, which harbor both *bla*_IMP-4_ and the *tmexCD2-toprJ2* efflux pump gene cluster, selection was performed on LB agar supplemented with 2  mg/L meropenem, 2  mg/L tigecycline, and 100  mg/L sodium azide. For plasmids pKP57-3 and pKP58-3, which carry *bla*_NDM-1_, *ant (2'')-Ia*, *qnrA1*, and *sul1*, transconjugants were selected on medium containing 2  mg/L meropenem, 10  mg/L gentamicin, and 100  mg/L sodium azide. The conjugation frequency was calculated as the number of transconjugants obtained per donor cell. The frequency of conjugation was determined by dividing the number of obtained transconjugants by the number of donor cells. Putative transconjugants were subjected to PCR amplification to confirm the presence of target resistance genes (Table 1), verifying plasmid transfer. Species identification of verified transconjugants was further confirmed using the VITEK 2 Compact System (bioMérieux, France).

**TABLE 1:** Primer sequences used for PCR detection of resistance genes in transconjugants

Name	Sequence
*tmexC2*-F	CTATTTGATCGCCGTGAC
*tmexC2*-R	GATCGAGTTGCTGGATAC
*tmexD2*-F	GCAACATCAACAACGAAAT
*tmexD2*-R	CAGGAACAGGAACATCAC
*toprJ2*-F	GACTGGCAGACCTTCATC
*toprJ2*-R	GACCGCTTCCTGGTAATC
*bla*_IMP-4_-F	ATTAAGCCACTCTATTCC
*bla*_IMP-4_-R	AGCAAGTTATCTGTATTCT
*bla*_NDM-1_-F	CCAACGGTGATATTGTCA
*bla*_NDM-1_-R	CAGCACACTTCCTATCTC
*ant(2'')-Ia*-F	CAGGTCACATTGATACAC
*ant(2'')-Ia*-R	ACTTCATCGGCATAGTAA

### Phylogenetic analysis

All publicly available genomes of *K. quasipneumoniae* subsp. *quasipneumoniae* were downloaded from the NCBI RefSeq database (accessed on 25 November 2024). Assembly quality was evaluated using QUAST v5.2.0, and genomes exhibiting incomplete assemblies or poor contiguity metrics were excluded from subsequent analyses (26). To eliminate redundant genomes and maintain representative genetic diversity, assemblies were dereplicated with Assembly Dereplicator (https://github.com/rrwick/Assembly-Dereplicator) using a dereplication distance cutoff of 0.001. For comparative genomic analysis, Snippy v4.6.0 (https://github.com/tseemann/snippy) was used to identify and align core-genome single-nucleotide polymorphisms (SNPs) against the reference strain *K. quasipneumoniae* subsp. *similipneumoniae* ATCC 700603 (accession: GCF_003181175.1). To mitigate the confounding effects of recombination, the alignment was analyzed using Gubbins v3.3.0 (27). The filtered alignment was subsequently processed with SNP-sites v2.6.0 to extract nonrecombinant core SNP positions (28). A maximum likelihood phylogenetic tree was inferred using FastTree v2.1, employing the GTR + Gamma model to model nucleotide substitution and rate heterogeneity across sites (29). The resulting phylogeny was visualized and annotated in the Interactive Tree of Life online platform (30).

### Statistical analysis

All statistical analyses were performed using GraphPad Prism software (v9.5.1; GraphPad Software, San Diego, CA, USA). Comparisons among multiple groups were analyzed by one-way analysis of variance, and when appropriate, significant differences between individual groups were further identified using Tukey’s multiple comparison test. Statistical significance was defined as a *P*  <  0.05. Experimental data are expressed as the mean  ±  SEM, and graphical error bars reflect the corresponding variability.

## RESULTS

### Clinical origins and genomic characteristics of the ST978 *Klebsiella quasipneumoniae* subsp. *quasipneumoniae* isolates

Four MDR *Klebsiella quasipneumoniae* subsp. *quasipneumoniae* isolates, named KP55, KP57, KP58, and KP79, were recovered from four hospitalized patients in a tertiary teaching hospital in China between January and May 2024. KP55 was isolated from the blood of a 72-year-old male patient who had been admitted to the neurology ward due to an acute ischemic stroke complicated by aspiration pneumonia. The patient had a medical history of hypertension and atrial fibrillation. On day 10 of hospitalization, he developed persistent fever and leukocytosis. Despite empirical treatment with meropenem and linezolid, his condition progressed to septic shock, and he died on day 37. KP57 was obtained from a sputum sample collected from a 65-year-old female patient with chronic obstructive pulmonary disease. She had been admitted to the respiratory medicine department for an acute exacerbation and secondary bacterial pneumonia. The patient had recently completed a course of ceftriaxone and azithromycin. After initial clinical improvement, she developed ventilator-associated pneumonia and deteriorated rapidly. Although antibiotic therapy was escalated to tigecycline and polymyxin B, her condition continued to worsen, and she was transferred to the intensive care unit with multi-organ dysfunction. KP58 was recovered from the tip of a central venous pressure catheter from a 70-year-old male patient admitted to the cardiology department for acute myocardial infarction. He underwent percutaneous coronary intervention and subsequently developed a catheter-related bloodstream infection 7 days after the procedure. Empirical broad-spectrum antibiotics, including piperacillin–tazobactam and amikacin, were administered, but the infection remained unresponsive. The patient developed persistent bacteremia and died of cardiac failure complicated by sepsis. KP79 was isolated from the cerebrospinal fluid of a 58-year-old male patient with a history of epilepsy and prior neurosurgery. He was admitted to the neurology department with clinical signs of meningitis. Lumbar puncture revealed elevated leukocyte count and reduced glucose levels. Empirical treatment with vancomycin and meropenem was initiated, but his condition continued to deteriorate. Despite the addition of polymyxin B, the patient developed hydrocephalus requiring external ventricular drainage and was eventually discharged in a persistent vegetative state at the request of his family.

All four isolates displayed a non-hypermucoid phenotype, as evidenced by the absence of viscous filament formation in the string test. Preliminary species identification based on MALDI-TOF MS analysis incorrectly classified the isolates as *K. pneumoniae*. Subsequent whole-genome sequencing clarified their taxonomic placement as *K. quasipneumoniae* subsp. *quasipneumoniae*. Genomic assemblies revealed genome lengths between 5.68 and 5.87 Mb. Multilocus sequence typing identified all isolates as belonging to sequence type (ST) ST978, characterized by the allelic profile: *gapA* (17), *infB* (55), *mdh* (39), *pgi* (20), *phoE* (117), *rpoB* (18), and *tonB* (156). Capsule locus analysis assigned the isolates to KL103, consistent with the presence of the wzi 301 allele.

### Antimicrobial resistance profiles of the ST978-KL103 *K. quasipneumoniae* subsp. *quasipneumoniae* isolates

All four ST978-KL103 *K. quasipneumoniae* subsp. *quasipneumoniae* isolates exhibited an XDR phenotype, characterized by high-level resistance to multiple clinically important classes of antibiotics (Table 2). Antimicrobial susceptibility testing revealed that all isolates were resistant to carbapenems, aminoglycosides, fluoroquinolones, and β-lactam/β-lactamase inhibitor combinations, including ampicillin-sulbactam, piperacillin-tazobactam, and ceftazidime-avibactam. In addition, resistance was observed against aztreonam and a wide range of cephalosporins, including cefuroxime and ceftazidime. The isolates also displayed resistance to tigecycline and trimethoprim/sulfamethoxazole. Polymyxin B remained the only agent to which all four isolates were susceptible, with MICs ranging from 0.5 to 1 µg/mL.

**TABLE 2:** Antimicrobial susceptibility profiles of ST978-KL103 *K. quasipneumoniae* subsp. *quasipneumoniae* isolates^*a*^

Antimicrobial agent	MIC (μg/mL, S/I/R)			
	KP55	KP57	KP58	KP79
Ampicillin/sulbactam	>32/16 (R)	>32/16 (R)	>32/16 (R)	>32/16 (R)
Piperacillin/tazobactam	32/4 (R)	64/4 (R)	64/4 (R)	32/4 (R)
Aztreonam	>16 (R)	>16 (R)	>16 (R)	>16 (R)
Cefuroxime	>16 (R)	>16 (R)	>16 (R)	>16 (R)
Ceftazidime	>128 (R)	>128 (R)	>128 (R)	>128 (R)
Ceftazidime-avibactam	>16/4 (R)	>16/4 (R)	>16/4 (R)	>16/4 (R)
Imipenem	>16 (R)	>16 (R)	>16 (R)	>16 (R)
Meropenem	>16 (R)	>16 (R)	>16 (R)	>16 (R)
Amikacin	>64 (R)	>64 (R)	>64 (R)	>64 (R)
Gentamicin	>16 (R)	>16 (R)	>16 (R)	>16 (R)
Levofloxacin	>8 (R)	>8 (R)	>8 (R)	>8 (R)
Moxifloxacin	>2 (R)	>2 (R)	>2 (R)	>2 (R)
Tigecycline	>32 (R)	>32 (R)	>32 (R)	>32 (R)
Trimethoprim/sulfamethoxazole	>4/76 (R)	>4/76 (R)	>4/76 (R)	>4/76 (R)
Polymyxin B	1 (S)	1 (S)	1 (S)	0.5 (S)

^*a*^ R, resistant; I, intermediate; S, sensitive.

### Chromosomal and plasmid-mediated antimicrobial resistance in ST978-KL103 *K. quasipneumoniae* subsp. *quasipneumoniae* isolates

To investigate the genomic basis of antimicrobial resistance and virulence, we analyzed the chromosomal and plasmid features of four ST978-KL103 *K. quasipneumoniae* subsp. *quasipneumoniae* isolates (KP55, KP57, KP58, and KP79). All four isolates harbored chromosomes of similar size, ranging from 5.46 to 5.48 Mb, with a guanine–cytosine content of approximately 57.8%. Chromosomally encoded resistance genes included the intrinsic β-lactamase *bla*_OKP-A-9_, *fosA*, *fosA6*, and the efflux pump components o*qxA* and *oqxB*, with *sul2* additionally identified in KP55 and KP79 (Table 3). No chromosomally encoded virulence factors were detected. All four ST978-KL103 isolates carried multiple plasmids (3–4 per strain), among which a large IncU-type plasmid (~360–380 kb) was detected in each isolate (designated pKP55-1, pKP57-1, pKP58-1, and pKP79-1). These IncU plasmids consistently carried a broad array of resistance determinants, including *bla*_IMP-4_, the plasmid-borne tigecycline resistance gene cluster *tmexCD2-toprJ2*, as well as genes encoding aminoglycoside-modifying enzymes, and resistance to macrolides, quinolones, and chloramphenicol.

**TABLE 3:** Genomic characteristics and identified antimicrobial resistance genes in ST978-KL103 *K. quasipneumoniae* subsp. *quasipneumoniae* isolates^*a*^

Strain	Name	Size (bp)	Guanine–cytosine content (%)	Plasmid type	Resistance genes	Virulence genes
KP55	Chromosome	5,476,137	57.80	/	*bla*_OKP-A-9_, *fosA6*, *fosA*, *oqxB*, *oqxA*, *sul2*	/
	pKP55-1	379,043	49.10	IncU	*aph(6)-Id*, *aph(3'')-Ib*, *aac(6')-Ib3*, *aac(3)-IId*, *bla*_SFO-1_, *bla*_IMP-4_, *bla*_TEM-1B_, *mph(A*), *catB3*, *qnrS1*, *tmexC2, tmexD2, toprJ2*	/
	pKP55-2	140,886	50.40	IncFIB	/	/
	pKP55-3	22,728	50.23	IncFIA	/	/
KP57	Chromosome	5,464,787	57.81	/	*bla*_OKP-A-9_, *fosA6*, *fosA*, *oqxB*, *oqxA*, *sul2*	/
	pKP57-1	359,678	49.13	IncU	*aph(6)-Id*, *aph(3'')-Ib*, *aac(6')-Ib3*, *aac(3)-IId*, *bla*_SFO-1_, *bla*_IMP-4_, *bla*_OXA-1_, *bla*_TEM-1B_, *mph(A*), *catB3*, *qnrS1*, *tmexC2, tmexD2, toprJ2*	/
	pKP57-2	142,147	50.47	IncFIB	/	/
	pKP57-3	154,126	51.05	IncC	*ant(2'')-Ia*, *bla*_NDM-1_, *qnrA1*, *sul1*	/
KP58	Chromosome	5,462,944	57.81	/	*bla*_OKP-A-9_, *fosA6*, *fosA*, *oqxB*, *oqxA*, *sul2*	/
	pKP58-1	359,781	49.13	IncU	*aph(6)-Id*, *aph(3'')-Ib*, *aac(6')-Ib3*, *aac(3)-IId*, *bla*_SFO-1_, *bla*_IMP-4_, *bla*_OXA-1_, *bla*_TEM-1B_, *mph(A*), *catB3*, *qnrS1*, *tmexC2, tmexD2, toprJ2*	/
	pKP58-2	141,243	50.45	IncFIB	/	/
	pKP58-3	154,101	51.05	IncC	*ant(2'')-Ia*, *bla*_NDM-1_, *qnrA1*, *sul1*	/
KP79	Chromosome	5,465,603	57.80	/	*bla*_OKP-A-9_, *fosA6*, *fosA*, *oqxB*, *oqxA*, *sul2*	/
	pKP79-1	381,040	49.05	IncU	*aph(6)-Id*, *aph(3'')-Ib*, *aac(6')-Ib3*, *aac(3)-IId*, *bla*_SFO-1_, *bla*_IMP-4_, *bla*_TEM-1B_, *mph(A*), *catB3*, *qnrS1*, *tmexC2, tmexD2, toprJ2*	/
	pKP79-2	142,416	50.46	IncFIB	/	/
	pKP79-3	13,322	49.45	/	/	/
	pKP79-4	14,508	53.98	/	/	/

^*a*^ “/” indicates not detected or not present.

**Fig 1:**
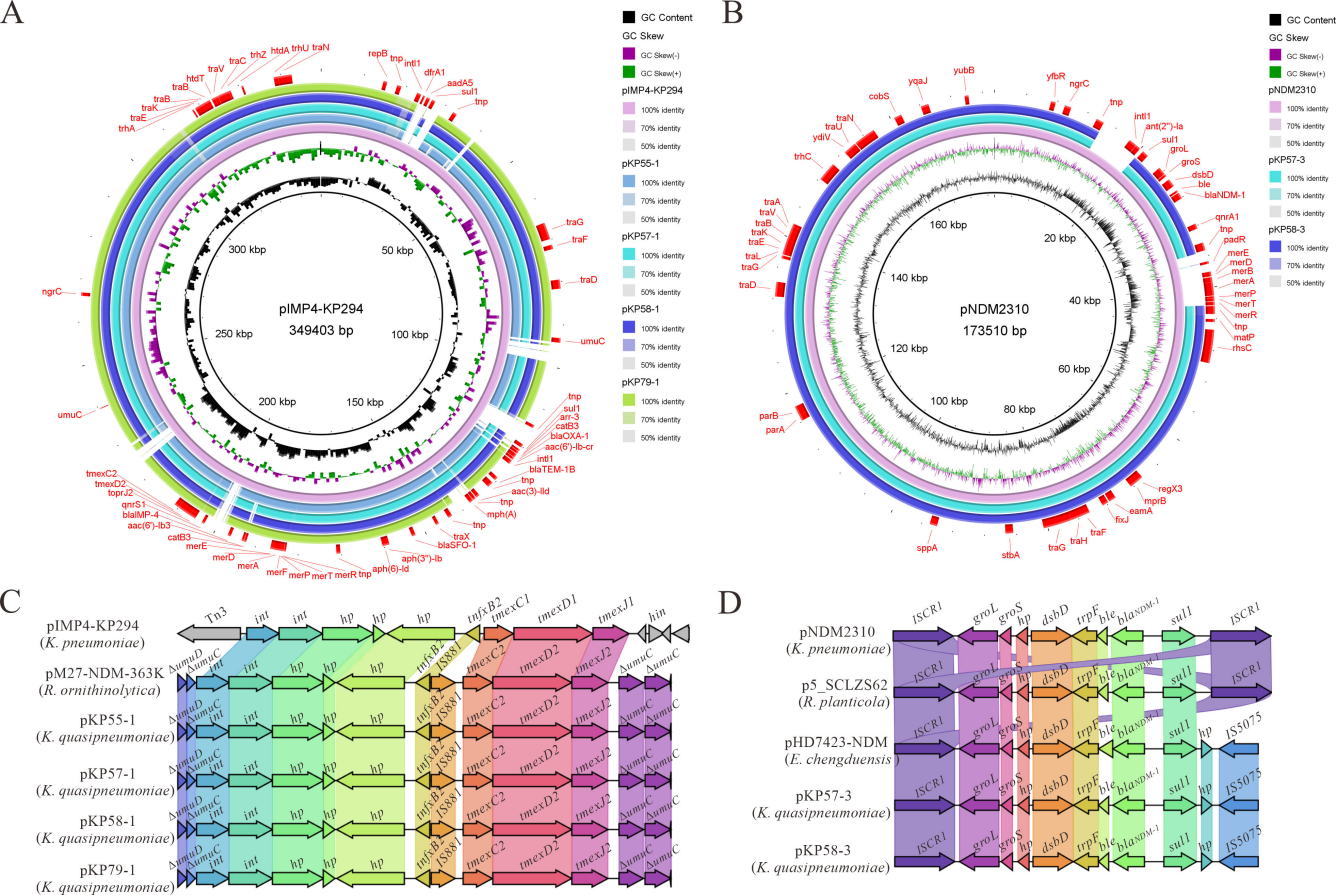
Comparative genetic analysis of plasmids in ST978-KL103 *K. quasipneumoniae* subsp. *quasipneumoniae* isolates. (**A**) Comparison of IncU-type multidrug resistance plasmids pKP55-1, pKP57-1, pKP58-1, and pKP79-1 with reference plasmid pIMP4-KP294 (*K. pneumoniae*, GenBank: CP083446.1). (**B**) Circular comparison of IncC-type plasmids pKP57-3 and pKP58-3 with pNDM2310 (*K. pneumoniae*, GenBank: CP138530.1). (**C**) Linear alignment of the *tmexCD2-toprJ2* gene cluster from pKP55-1, pKP57-1, pKP58-1, and pKP79-1, alongside reference plasmids pIMP4-KP294 and pM27-NDM-363K (*Raoultella ornithinolytica*, CP130154.1). (**D**) Linear comparison of the blaNDM-1 gene cluster in IncC plasmids pKP57-3 and pKP58-3 with reference plasmids pNDM2310 (*K. pneumoniae*, CP138530.1), p5_SCLZS62 (*Raoultella planticola*, CP082173.1), and pHD7423-NDM (*Enterobacter chengduensis*, ON209153.1).

Comparative analysis revealed a high degree of sequence similarity between these IncU plasmids and the previously reported multidrug resistance plasmid pIMP4-KP294 from *K. pneumoniae* (GenBank accession: CP083446.1; Fig. 1A). Additionally, as shown in Fig. 1B, KP57 and KP58 carried an extra IncC-type plasmid (pKP57-3 and pKP58-3, ~154 kb), which was absent in KP55 and KP79. These plasmids harbored *bla*_NDM-1_, *ant (2'')-Ia*, *qnrA1* (quinolone resistance). Comparative genomic analysis revealed that these IncC plasmids shared high similarity with the *bla*_NDM-1_-encoding plasmid pNDM2310 from *K. pneumoniae* (GenBank accession: CP138530.1). Structural comparisons of resistance gene clusters provided further insights into their genetic contexts. As illustrated in Fig. 1C, the *tmexC2D2-toprJ2* gene cluster in the IncU plasmids was flanked by *umuC* and *umuD* genes, forming a conserved structure: *IS881-tmexC2-tmexD2-toprJ2-umuC*. Linear analysis of the *bla*_NDM-1_ region in IncC plasmids (Fig. 1D) showed that the resistance island was flanked by ISCR1 and IS5075, forming the conserved structure: *ISCR1-groL-groS-dsbD-trpF-ble-bla*_NDM-1_-*sul1-IS5075*, a well-known mobile element associated with carbapenem resistance dissemination.

### Transferability of multidrug resistance plasmids in ST978-KL103 isolates

To evaluate the transferability of resistance plasmids identified in ST978-KL103 *K. quasipneumoniae* subsp. *quasipneumoniae* isolates, conjugation assays were performed using *E. coli* J53 as the recipient. The large IncU-type plasmids (pKP55-1, pKP57-1, pKP58-1, and pKP79-1), which co-harbored *bla*_IMP-4_ and the *tmexCD2-toprJ2* tigecycline resistance cluster, were successfully transferred into *E. coli* J53. The average conjugation frequencies ranged from 1.2 × 10^−6^ to 2.3 × 10^−6^ transconjugants per donor cell (Table 4). In addition, the IncC-type plasmids pKP57-3 and pKP58-3, which carried *bla*_NDM-1_, *ant(2'')-Ia*, *qnrA1*, and *sul1*, were also transferable to *E. coli* J53. Although the transfer frequencies were slightly lower (ranging from 6.5 × 10^−8^ to 1.1 × 10^−7^), successful transconjugants were consistently recovered in all independent replicates, and PCR confirmed the presence of *bla*_NDM-1_ in recipient strains.

**TABLE 4:** Conjugation frequencies of resistance plasmids from ST978-KL103 *K. quasipneumoniae* subsp. *quasipneumoniae* isolates

Plasmid	Resistance genes	No. of independent determinations	Conjugation frequency (mean)	Range
pKP55-1	*bla*_IMP-4_ *, tmexC2, tmexD2, toprJ2*	3	1.8 × 10⁻⁶	1.2 × 10⁻⁶ to 2.3 × 10⁻⁶
pKP57-1	*bla*_IMP-4_ *, tmexC2, tmexD2, toprJ2*	3	2.0 × 10⁻⁶	1.5 × 10⁻⁶ to 2.5 × 10⁻⁶
pKP58-1	*bla*_IMP-4_ *, tmexC2, tmexD2, toprJ2*	3	2.3 × 10⁻⁶	1.7 × 10⁻⁶ to 2.9 × 10⁻⁶
pKP79-1	*bla*_IMP-4_ *, tmexC2, tmexD2, toprJ2*	3	1.6 × 10⁻⁶	1.0 × 10⁻⁶ to 2.0 × 10⁻⁶
pKP57-3	*bla*_NDM-1_ *, ant(2'')-Ia, qnrA1, sul1*	3	8.0 × 10⁻⁸	6.5 × 10⁻⁸ to 1.1 × 10⁻⁷
pKP58-3	*bla*_NDM-1_ *, ant(2'')-Ia, qnrA1, sul1*	3	7.5 × 10⁻⁸	5.0 × 10⁻⁸ to 1.0 × 10⁻⁷

### Phenotypic characterization and virulence assessment of ST978-KL103 *K. quasipneumoniae* subsp. *quasipneumoniae* isolates

**Fig 2:**
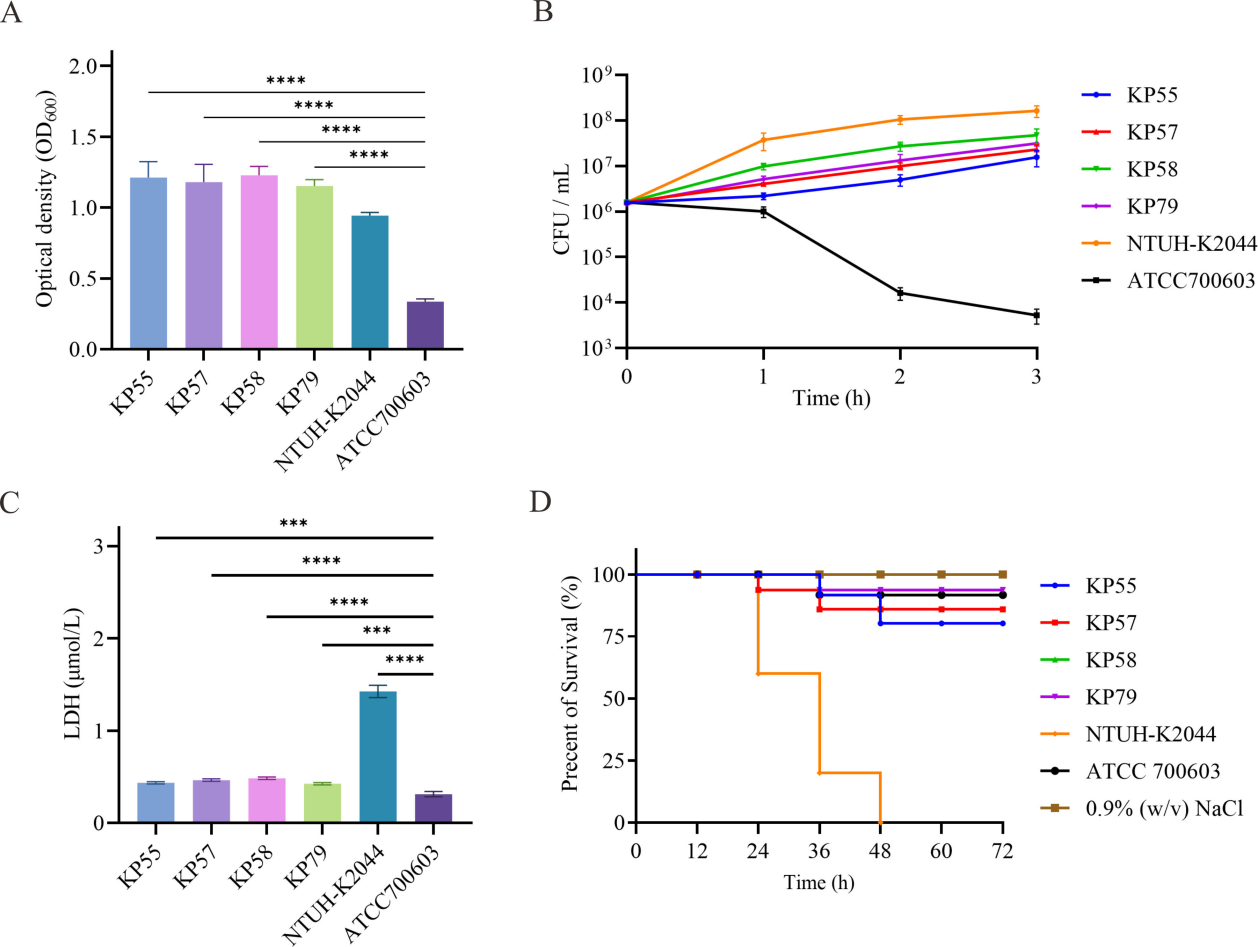
Virulence phenotypes of ST978-KL103 *K. quasipneumoniae* subsp. *quasipneumoniae* isolates. (**A**) Biofilm formation quantified by crystal violet staining after 24 h of static incubation at 37°C. (**B**) Serum resistance assay. (**C**) Cytotoxicity measured by LDH release from A549 cells after 6 h of infection. (**D**) *Galleria mellonella* survival curves over 72 h following infection with 1 × 10⁶ CFU per larva. ***P < 0.001 and ****P < 0.0001.

Genomic analysis revealed that all isolates lacked key virulence-associated loci, including yersiniabactin (*ybt*), aerobactin (*iuc*), salmochelin (*iro*), *rmpA*/*rmpA2*, and colibactin (*clb*). Phenotypic assays were performed to further characterize the virulence potential of the ST978-KL103 *K. quasipneumoniae* subsp. *quasipneumoniae* isolates, including biofilm formation, serum survival, cytotoxicity, and *G. mellonella* infection model. As shown in Fig. 2A, all four ST978-KL103 isolates (KP55, KP57, KP58, and KP79) exhibited strong biofilm-forming capacity. Quantitative analysis using the crystal violet staining method demonstrated that the mean OD_600_ values for KP55, KP57, KP58, and KP79 were 1.211 ± 0.113, 1.179 ± 0.124, 1.228 ± 0.062, and 1.153 ± 0.045, respectively. These values were higher than the *K. quasipneumoniae* subsp. *similipneumoniae* strain ATCC 700603 (0.335 ± 0.020) and the hypervirulent strain NTUH-K2044 (0.944 ± 0.021).

Serum resistance was assessed by exposing bacterial suspensions to human serum over a 3 h period. As shown in Fig. 2B, all ST978-KL103 isolates demonstrated strong resistance to complement-mediated killing. At 3 h post-incubation, CFU counts of KP55, KP57, KP58, and KP79 increased by approximately 1–2 log units relative to their initial inocula, with KP58 reaching up to 6.0 × 10⁷ CFU/mL. In contrast, the ATCC 700603 exhibited rapid killing, with CFU counts decreasing to below 10^4^ CFU/mL by 3 h. The hypervirulent strain NTUH-K2044 showed a pronounced increase in survival, reaching over 1.9 × 10^8^ CFU/mL. These results indicate that ST978-KL103 isolates possess strong serum resistance, comparable to NTUH-K2044. Cytotoxicity was evaluated by measuring LDH release from A549 cells at 6 h post-infection. As shown in Fig. 2C, all ST978-KL103 isolates induced moderate LDH release, with mean values of 0.42 ± 0.01 µmol/L (KP55) to 0.49 ± 0.01 µmol/L (KP58). These levels were higher than ATCC 700603 (0.31 ± 0.03 µmol/L, *P* < 0.001) but significantly lower than NTUH-K2044 (1.42 ± 0.10 µmol/L, *P* < 0.0001). Virulence was further assessed using the *G. mellonella* infection model. As shown in Fig. 2D, larvae infected with ST978-KL103 isolates (1 × 10⁶ CFU per larva) exhibited high survival rates, with over 80% of larvae surviving at 72 h. The survival rates of these groups were not statistically different from those of the ATCC 700603 reference strain or the saline (0.9% NaCl) controls.

### Phylogenetic analysis and global distribution of ST978 *K. quasipneumoniae* subsp. *quasipneumoniae* strains

**Fig 3:**
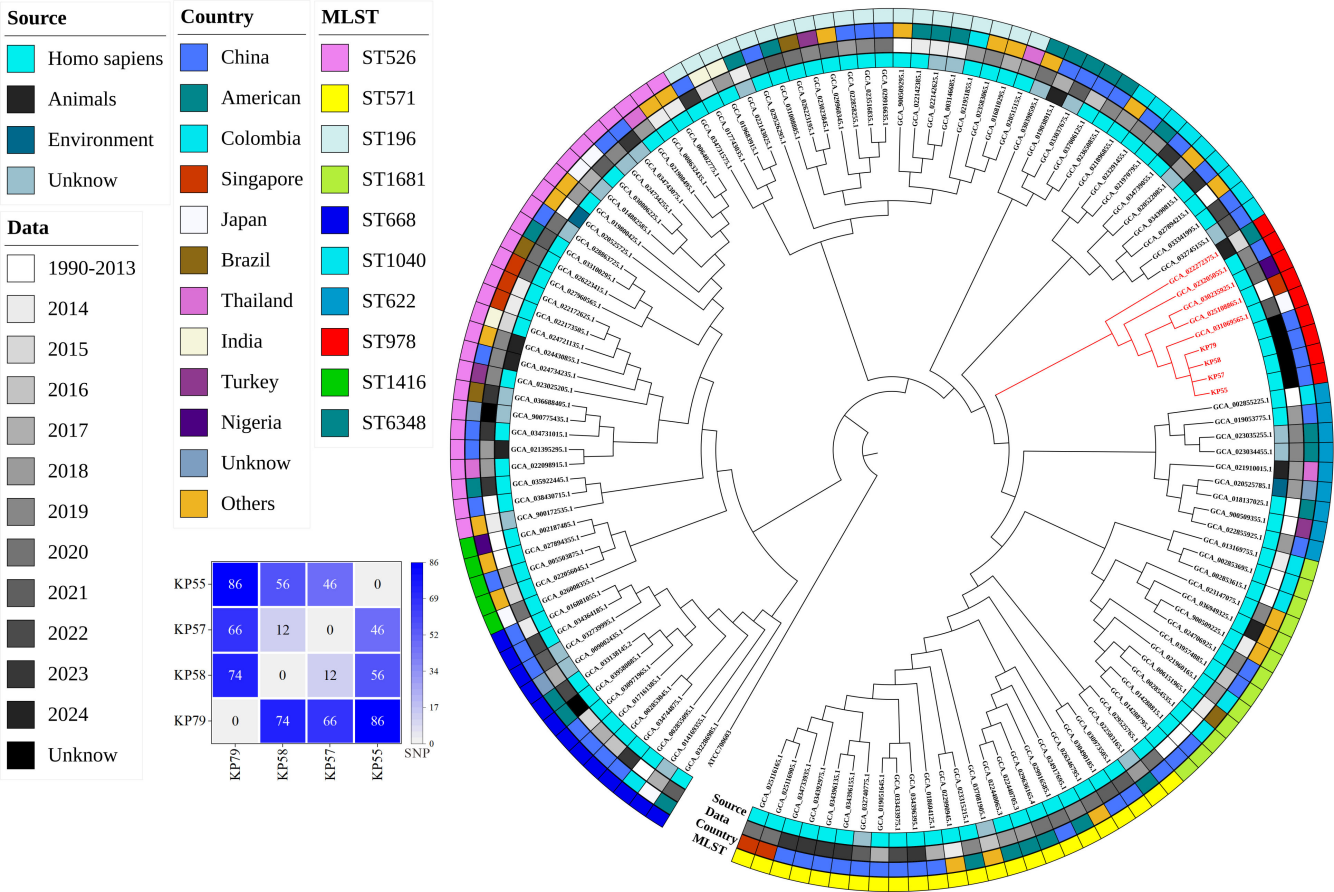
Phylogenetic analysis of *K. quasipneumoniae* subsp. *quasipneumoniae* isolates based on core-genome SNPs. A maximum-likelihood phylogenetic tree was constructed using 130 publicly available *K. quasipneumoniae* subsp. *quasipneumoniae* genomes and four ST978 isolates from this study (KP55, KP57, KP58, and KP79). Outer colored rings represent metadata, including source (host), country of origin, year of isolation, and MLST type. A distinct monophyletic clade (highlighted in red) corresponds to all known ST978 isolates (*n* = 9). Inset heatmap: pairwise SNP distances among the four ST978 isolates from this study.

To explore the phylogenetic relationship and population structure of *K. quasipneumoniae* subsp. *quasipneumoniae* isolates, a core-genome SNP-based phylogenetic analysis was conducted using 130 *K*. *quasipneumoniae* genomes available in the NCBI RefSeq database, together with the four ST978-KL103 isolates obtained in the present study. As shown in Fig. 3, the analyzed strains demonstrated wide geographical and host diversity. The majority of isolates were collected from China (*n* = 53), followed by the United States (*n* = 21), Colombia (*n* = 8), Singapore (*n* = 6), Japan (*n* = 5), Brazil and Thailand (*n* = 4 each), Turkey and India (*n* = 3 each), and Nigeria (*n* = 2). Most isolates originated from human clinical sources (*n* = 104), with smaller numbers isolated from animals (*n* = 6) and environmental samples (*n* = 2). Multilocus sequence typing revealed 10 distinct STs) among the 130 genomes. The most prevalent STs were ST526 (*n* = 27), ST571 (*n* = 24), and ST196 (*n* = 20). Less common STs such as ST622, ST1040, and ST978 were infrequently reported. A total of nine ST978 isolates were identified, including the four sequenced in this study and five from public databases. These isolates formed a distinct monophyletic clade (Fig. 3, highlighted in red), separate from other STs, indicating close genetic relatedness and a likely clonal origin. The pairwise SNP distances among the four isolates from this study ranged from 12 to 86 SNPs (median = 61). Notably, KP57 and KP58 differed by only 12 SNPs, suggesting a recent common ancestor and potential hospital-based transmission. The overall phylogenetic structure indicated that the ST978 lineage possesses low intra-lineage genetic diversity. The earliest known ST978 genome was recorded in 2016 in San Diego, CA, USA, followed by subsequent detections in Singapore (2021), China (2022), Japan (2023), and Nigeria (2023).

### ST978 *K. quasipneumoniae* subsp. *quasipneumoniae* isolates harbor a highly diverse and alarming resistome

**Fig 4:**
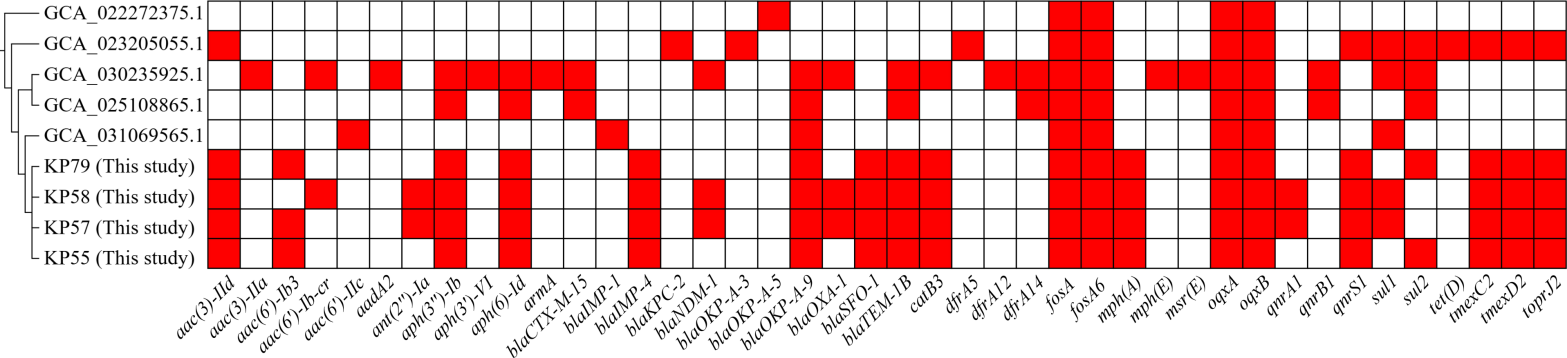
Resistome diversity among ST978 *K. quasipneumoniae* subsp. *quasipneumoniae* isolates. Heatmap showing the presence (red) or absence (white) of 42 antimicrobial resistance genes across nine ST978 isolates (five from public databases and four from this study).

To characterize the antimicrobial resistance potential of global ST978 *K. quasipneumoniae* subsp. *quasipneumoniae*, we analyzed the resistome profiles of all nine publicly available ST978 genomes, including the four clinical isolates from this study and five additional genomes retrieved from public databases. As shown in Fig. 4, these isolates collectively harbored a remarkably diverse array of resistance genes, spanning multiple antimicrobial classes, including last-resort agents such as carbapenems and tigecycline, as well as β-lactams, aminoglycosides, fluoroquinolones, macrolides, sulfonamides, and tetracyclines. In total, over 40 distinct resistance genes were identified across the ST978 isolates. These included carbapenemase genes such as *bla*_IMP-1_, *bla*_IMP-4_, *bla*_NDM-1_, and *bla*_KPC-2_, as well as extended-spectrum β-lactamases such as *bla*_CTX-M-15_, *bla*_SFO-1_, *bla*_OXA-1_, and *bla*_TEM-1B_. A wide range of aminoglycoside-modifying enzymes [*aac(3)-IId*, *aac(6')-Ib3*, *aph(3'')-Ib*, and *ant(2'')-Ia*], fluoroquinolone resistance genes (*qnrA1*, *qnrB1*, and *qnrS1*), macrolide resistance determinants [*mph(A)*, *mph(E)*, and *msr(E)*], and efflux pump-associated gene clusters (*oqxAB*, *tmexC2D2-toprJ2*) were also detected. Additionally, some isolates carried *armA*, *sul1*, *sul2*, *tet(D)*, and *catB3*. Importantly, all four ST978 isolates from this study (KP55, KP57, KP58, and KP79) consistently harbored the plasmid-borne tigecycline resistance cluster *tmexC2D2-toprJ2*, a key determinant associated with reduced efficacy of one of the last-resort antibiotics. In contrast, among the five ST978 genomes from public databases, only one isolate carried this resistance cluster. This difference underscores the unique and potentially escalating resistance capacity of the ST978 isolates identified in our study, suggesting that this lineage may be evolving toward an extensively drug-resistant phenotype. Furthermore, uncommon but concerning combinations of resistance determinants were identified in public genomes, such as the co-occurrence of *bla*_KPC-2_ and *bla*_NDM-1_, or *bla*_CTX-M-15_ with *armA* and *tet(D)*, indicating a high capacity for gene acquisition and resistome plasticity within the ST978 lineage.

## DISCUSSION

This study presents the comprehensive characterization of *K. quasipneumoniae* subsp. *quasipneumoniae* ST978-KL103 isolates co-harboring both *tmexC2D2-toprJ2* and *bla*_IMP-4_, an emerging sequence type increasingly implicated in severe infections. Although historically overshadowed by the more virulent *K. pneumoniae* strains, our findings suggest that ST978-KL103 *K. quasipneumoniae* subsp. *quasipneumoniae* may be evolving into a clinically significant, high-risk clonal lineage characterized by extensive drug resistance, cryptic virulence, and horizontal gene transfer potential.

Accurate species identification remains a fundamental challenge in clinical microbiology, particularly within the *K. pneumoniae* complex, where closely related taxa often exhibit overlapping phenotypic features (8, 31). In this study, all four ST978 isolates were initially misidentified as *K. pneumoniae* by MALDI-TOF MS, reflecting a common limitation in routine diagnostics that lack the resolution to distinguish among cryptic species. Such misclassification can compromise epidemiological surveillance and delay appropriate clinical management, especially when the misidentified strains harbor uncommon resistance or virulence traits.

All four ST978-KL103 isolates exhibited XDR phenotypes and were recovered from patients with life-threatening conditions, including bloodstream infection, ventilator-associated pneumonia, and meningitis, each of which resulted in either mortality or severe sequelae. Notably, the convergence of resistance elements is typically associated with high-risk Enterobacterales, such as *bla*_IMP-4_, *bla*_NDM-1_, and *tmexCD2-toprJ2*, within a *K. quasipneumoniae* species (7, 32). Despite their close phylogenetic relationship, *K. quasipneumoniae* has traditionally been considered less clinically relevant, partly due to its rarity and presumed lower virulence (7, 33). However, the ST978-KL103 isolates were associated with invasive infections and poor clinical outcomes and exhibited a resistance profile that effectively nullifies most available antibiotics. The ability of ST978 to accumulate and maintain multiple carbapenemase genes and a plasmid-encoded tigecycline efflux pump on conjugative plasmids suggests that selective pressure in hospital environments may be driving the emergence of such multi-resistant clones across species boundaries.

All isolates harbored intrinsic chromosomal genes (*bla*_OKP-A-9_, *oqxAB*, *fosA*) conferring baseline resistance, but their XDR phenotype was largely attributable to plasmid-borne determinants. Each strain carried a large conjugative IncU-type plasmid co-harboring *bla*_IMP-4_ and the tigecycline resistance cluster *tmexCD2-toprJ2*. Additionally, KP57 and KP58 carried an extra IncC-type plasmid encoding *bla*_NDM-1_, *ant(2'')-Ia*, *qnrA1*, and *sul1*. The identification of *tmexCD2-toprJ2* in all four isolates is particularly noteworthy. These findings raise broader concerns about the role of *K. quasipneumoniae* subsp. *quasipneumoniae* as a silent reservoir of multidrug resistance, capable of acting as both recipient and donor in the microbial resistome. Compounding the therapeutic challenge posed by this resistome is the limited diagnostic resolution of conventional identification methods.

In addition to their extensive resistance profile, the ST978-KL103 isolates demonstrated phenotypic characteristics commonly associated with bacterial persistence and evasion of host immune responses. These included strong biofilm-forming capacity and resistance to serum killing, despite the absence of well-characterized virulence loci. RND-type efflux systems are known to confer tolerance to antimicrobial stress and have been shown to enhance resistance to host innate immune defenses, thereby contributing to virulence in *K. pneumoniae* (34). Although direct evidence linking the *tmexCD2-toprJ2* efflux pump clusters to these traits is currently lacking, the presence of this system in all ST978 isolates may promote biofilm formation and enhance bacterial survival within the host, thereby contributing to persistent infection under hostile conditions. Serum resistance is largely determined by the structural and functional properties of surface components, including the capsule, lipopolysaccharides, and outer membrane proteins that interfere with complement recognition and attack (35). The capsule, in particular, acts as a physical barrier that restricts complement deposition and activation (35, 36). Although the specific structural features of the KL103 capsular polysaccharide remain uncharacterized, its composition may contribute to the observed serum resistance by limiting complement-mediated attack on the bacterial surface. Biofilm formation is particularly relevant in clinical settings, as it facilitates bacterial adherence to abiotic surfaces, including medical devices, and provides protection against both antimicrobial agents and host immune defenses (37). The co-occurrence of these traits in ST978-KL103 isolates suggests an evolutionary adaptation favoring persistence and environmental resilience rather than acute virulence. This phenotype may support prolonged colonization, increased antibiotic tolerance, and a higher risk of device-associated infections, thereby contributing to nosocomial transmission and poor clinical outcomes.

Phylogenetic analysis demonstrated that ST978 constitutes a monophyletic clade with limited genetic variation, a pattern indicative of recent clonal expansion. Its identification in geographically dispersed regions, including Asia, North America, and Africa, suggests international dissemination. The minimal SNP differences observed between certain isolates, particularly KP57 and KP58, provide evidence of recent nosocomial transmission, underscoring the importance of continuous genomic surveillance and stringent infection control measures. Although ST978 remains infrequently represented in public genomic repositories, its consistent association with extensive drug resistance, persistence-related phenotypes, and potential for horizontal gene transfer resembles the early evolutionary trajectory of high-risk clones such as ST11 (38). These findings indicate that ST978 *K. quasipneumoniae* subsp. *quasipneumoniae* is not only globally distributed but also carries a highly diverse and dynamic resistome. This includes transferable plasmids conferring resistance to multiple antimicrobial classes. Of particular concern is the detection of *tmexCD2-toprJ2* in all four clinical isolates from our study, a resistance determinant that shows a low prevalence among *Klebsiella* genomes (39). The convergence of genetic clonality, international spread, and accumulation of plasmid-mediated resistance determinants strongly suggests that ST978 is evolving into a high-risk clone with the potential to impact both clinical and environmental microbial ecosystems.

Our study has several limitations. The number of ST978 isolates was small and restricted to a single hospital, limiting the generalizability of our findings. The lack of environmental or colonization isolates also precludes a comprehensive understanding of transmission dynamics and potential reservoirs. Effective mitigation of this threat will require a comprehensive strategy that includes improved diagnostic methodologies capable of resolving species- and strain-level differences, expanded genomic surveillance to detect and track emerging lineages, and strengthened antimicrobial stewardship programs aimed at reducing the selective pressures that drive resistance evolution. Additionally, although *in vitro* and *in vivo* assays support the persistence and low acute virulence of these isolates, further studies using mammalian models are needed to validate their pathogenic potential in more complex host environments.

In conclusion, ST978-KL103 represents a previously underrecognized yet clinically significant lineage within *K. quasipneumoniae* subsp. *quasipneumoniae*. The combination of extensive antimicrobial resistance, plasmid-mediated gene transfer, and persistence-associated traits confers a high potential for nosocomial dissemination and therapeutic failure. These characteristics suggest that ST978-KL103 may be emerging as a cryptic high-risk clone.

## Data Availability

The genome sequences in this study have been submitted to NCBI GenBank under accession number PRJNA1308511.
